# Coupling AFM, DSC and FT-IR towards Elucidation of Film-Forming Systems Transformation to Dermal Films: A Betamethasone Dipropionate Case Study

**DOI:** 10.3390/ijms23116013

**Published:** 2022-05-27

**Authors:** Mirjana D. Timotijević, Tanja Ilić, Bojan Marković, Danijela Randjelović, Nebojša Cekić, Ines Nikolić, Snežana Savić, Ivana Pantelić

**Affiliations:** 1 Department of Pharmaceutical Technology and Cosmetology, Faculty of Pharmacy, University of Belgrade, Vojvode Stepe 450, 11221 Belgrade, Serbia; timotijevic.mirjana@gmail.com (M.D.T.); tanja.ilic@pharmacy.bg.ac.rs (T.I.); ines.nikolic@pharmacy.bg.ac.rs (I.N.); snezana.savic@pharmacy.bg.ac.rs (S.S.); ivana.pantelic@pharmacy.bg.ac.rs (I.P.); 2 Department of Pharmaceutical Chemistry, Faculty of Pharmacy, University of Belgrade, Vojvode Stepe 450, 11221 Belgrade, Serbia; 3 Centre of Microelectronic Technologies, Institute of Chemistry, Technology and Metallurgy, University of Belgrade, Njegoševa 12, 11000 Belgrade, Serbia; danijela@nanosys.ihtm.bg.ac.rs; 4 Faculty of Technology, University of Niš, Bulevar Oslobođenja 124, 16000 Leskovac, Serbia; nesafarm@icloud.com; 5 DCP Hemigal, Tekstilna 97, 16000 Leskovac, Serbia

**Keywords:** hydrophobic polymethacrylate copolymers, hydroxypropyl cellulose, lipophilic drug, viscosity, porcine ear epidermis

## Abstract

Polymeric film-forming systems have emerged as an esthetically acceptable option for targeted, less frequent and controlled dermal drug delivery. However, their dynamic nature (rapid evaporation of solvents leading to the formation of thin films) presents a true characterization challenge. In this study, we tested a tiered characterization approach, leading to more efficient definition of the quality target product profiles of film-forming systems. After assessing a number of physico-chemico-mechanical properties, thermal, spectroscopic and microscopic techniques were introduced. Final confirmation of betamethasone dipropionate-loaded FFS biopharmaceutical properties was sought via an in vitro skin permeation study. A number of applied characterization methods showed complementarity. The sample based on a combination of hydrophobic Eudragit^®^ RS PO and hydroxypropyl cellulose showed higher viscosity (47.17 ± 3.06 mPa·s) and film thickness, resulting in sustained skin permeation (permeation rate of 0.348 ± 0.157 ng/cm^2^ h), and even the pH of the sample with Eudragit^®^ NE 30D, along with higher surface roughness and thermal analysis, implied its immediate delivery through the epidermal membrane. Therefore, this study revealed the utility of several methods able to refine the number of needed tests within the final product profile.

## 1. Introduction

In recent years, conventional drug dosage forms for topical application (ointments, creams, etc.) have offered limited advancements with regard to (trans)dermal delivery. On the other hand, adherence to such therapy protocols is particularly low and often requires frequent and messy drug application [1]. High-application frequency due to the low substantivity of semi-solid formulations, wherein up to 90% of the active substance is removed from the skin by clothing or by contact with other surfaces, negatively affects patient adherence and treatment efficacy [2]. Therefore, film-forming systems (FFSs) have emerged as an esthetically acceptable option for targeted, less frequent and controlled drug delivery.

These alternative dosage forms defined as non-solid, i.e., intermediate between transdermal patches and semisolid dosage forms, produce a film after application on the skin. By maintaining a close contact with the skin for prolonged periods of time, FFSs enable less frequent dosing and improve patient compliance [3].

Upon application, rapid evaporation of solvent(s) occurs, leaving a discrete in situ formed film on the skin surface, which is often imperceptible to the patient. Taking into consideration several aspects of prospective topical drug delivery systems (manufacturing costs, product quality, patient acceptability, etc.), FFS based on rationally selected film-forming polymers and conventional volatile solvents are expected to gradually increase their presence on the market [4,5].

Currently, several patented film-forming technologies based on polymers for dermal/transdermal drug delivery, such as Liqui-Patch^®^ (Epinamics GmbH, Berlin, Germany), Medspray^®^ (MedPharm Ltd., Guildford, UK) and Durapeel^®^ (Crescita Therapeutics Inc., Mississauga, ON, Canada) are available on the market [6,7,8]. Evamist^®^ MTDS was the first metered-dose transdermal spray developed by Acrux Inc. (Melbourne, Australia), further commercialized in 2016 under the Lenzetto^®^ brand, for transdermal delivery of estradiol in the treatment of hot flushes commonly associated with menopause [9]. Another product developed by Acrux, Axiron^®^ topical solution (Eli Lilly and Co., Indianapolis, USA), was the first sprayable film-forming solution approved by the FDA in 2010 for transdermal delivery of testosterone, which is used for hormonal replacement therapy in male hypogonadism (congenital or acquired) [10]. Another film-forming cutaneous solution present on the market is Lamisil Once^®^ 1% (GlaxoSmithKline Consumer Healthcare, Brentford, UK), which contains the antimycotic drug terbinafine hydrochloride for the treatment of athlete’s foot (*tinea pedis*) with a single application [11].

However, a more systematic approach to FFS assessment is essential to consistently deliver the intended performance of the product, ultimately leading to more marketed products.

The dynamic nature of FFS implies a need to define a tailored quality target product profile (QTPP), which can only be achieved by smart identification of critical quality attributes (CQAs) so that those product characteristics that have an impact on product quality can be further studied and controlled [12,13]. Apart from standard aspects relating to quality, safety and efficacy of preparations for cutaneous application, the so-called metamorphosis/transformation of FFS deserves careful assessment, considering its prospective influence on a number of properties (e.g., precipitation of the incorporated active pharmaceutical ingredient). Although the importance of testing metamorphosis is acknowledged by the latest EMA draft guideline [14], no single method appears to sufficiently characterize the entire process. Hence, this study encompassed a number of characterization methods, which were performed at four levels whenever applicable: with drug-loaded and unloaded FFS, in liquid form and upon film formation.

After assessing fundamental physicochemical, mechanical and tribological properties further reinforced with biocompatible and biosubstantive features of a series of FFSs [15], the refined number of samples was selected to enter the next stage of the study, aiming to assess precise film morphology and potential molecular interactions within such dynamic system. The dipropionate ester of betamethasone, herein chosen as the model active substance, is a potent corticosteroid frequently used in treatments of different types of dermatitis and chronic plaque psoriasis localized on elbows, knees, scalp and feet [16,17,18]. Betamethasone efficacy in the abovementioned indications might be improved with modern formulation strategies, such as targeted application of the polymeric FFS with enhanced substantivity at the target site, e.g., psoriasis plaques or the flexor surfaces of the forearms and legs. Furthermore, possibly reducing the application frequency of potent local corticosteroids and, subsequently, their undesirable effects, such systems could have a remarkable effect on patient compliance. Therefore, with this work, we aimed to contribute to the biophysical elucidation of the mechanism behind the FFS-to-film transition, possibly leading to enhanced drug dermal delivery of a lipophilic model compound—betamethasone dipropionate (BDP). After applying a set of physicomechanical methods (revealing the spreadability and drying time of the FFS, as well as the thickness of the generated films, their flexibility and basic sensory properties), thermal (differential scanning calorimetry, DSC) and spectroscopic (Fourier Transform Infrared Spectroscopy, FT-IR) methods were used as tools for the analysis of profound interactions that might occur upon FFS drying. Atomic force microscopy (AFM) was important for in depth film topography characterization and detection of potential drug crystallization during the phase transitions, whereas FFS viscosity and pH were monitored as a measure of preliminary stability during 6 months of storage. Because the drying process may cause phase transitions that affect not only physicochemical properties but also dermal availability [5] in the final stage, an in vitro permeation study was performed to compare the selected FFS and a commercial product comprising the same concentration of the lipophilic model drug, BDP.

## 2. Results and Discussion

### 2.1. Physicochemical Evaluation and Stability Screening of FFS

Besides the composition of the formulations (Table 1), the method of preparation may also influence FFS performance, especially properties relating to drug crystal formation during storage [12,19]. Although FFS preparation and storage (described in Section 3.2.) were designed to reduce this possibility, further investigations would fall into the scope of critical process parameters (CPP) and are outside the aims of this study. Viscosity, as one of the basic rheological parameters, and pH values in the selected time intervals (initially, after 3 months, and after 6 months) were screened for the long-term behavior and stability of such systems (Table 2).

Tested active samples had pH values in the range of 6.6 to 7.7, whereas the viscosity values spanned from 1.67 to 47.17 mPa·s. The observed differences in pH values cannot be considered statistically significant despite the fact that a slight decrease was recorded after 3 months (within 0.2 pH units) and after 6 months of storage (within 0.5 pH units). Furthermore, BDP is a neutral compound. Hence, the BDP-loaded samples showed somewhat higher pH values when compared to the placebo samples (6.9 ± 0.1, 7.5 ± 0.1, and 6.6 ± 0.1 for S1, S2 and S3, respectively). Formulation S2A was found to have the highest pH value of 7.7. pH values spanning the basic half of the pH scale could potentially provide more favorable dermal absorption [20]. The obtained findings imply that BDP-loaded FFSs would not produce either skin irritation or any other patient discomfort, as previously confirmed in vivo for corresponding samples without this model active substance [15].

It is common knowledge that the properties and the applied concentration of the chosen film-forming polymer(s) primarily influence the system’s viscosity. Therefore, considering prior knowledge of the polymers used in this study [21], samples S3 and S3A (comprising HPC at a concentration of 1%, *w*/*w*) were expectedly found to have the highest viscosity values, whereas samples S1 and S1A had the lowest viscosities due to the presence of Eudragit^®^ RS PO. Lower viscosity also implies faster lateral spreading of a product on the skin. Furthermore, the obtained viscosity values correlated well with the results obtained in our previous study [15], which encompassed an in vivo substantivity assay of ‘placebo’, i.e., drug-unloaded FFSs, wherein the level of skin substantivity was in the following order: S3 > S2 > S1. Therefore, early-stage rheology of FFS may provide a notion of bioadhesive and skin-retention properties of a polymeric formulation. Finally, the lack of statistically significant change in viscosity values over time implies sample stability despite the fact that a standard container-closure system was used throughout (glass vials with PP caps). Additionally, the generally low viscosities of the assessed FFSs make them suitable for more sophisticated packaging solutions, such as sprays or even metered dose containers, allowing for more precise drug dosing.

Considering the fact that the chosen FFS formulations stemmed from a previous study [15] and therefore differ in film-forming polymer type and/or concentration, the heterogeneous results presented within Table 3 were somewhat expected. The important thing to note is that the incorporation of BDP in the investigated FFS failed to induce a significant change of the monitored physicomechanical properties, meaning that BDP precipitation was unlikely to occur during product metamorphosis and subsequent film formation. A proof of this hypothesis was subsequently sought in the results of DSC, FT-IR and/or AFM measurements.

### 2.2. Thermal and Spectroscopic Analysis

Thermal and spectroscopic analyses are of particular importance in the early-stage development of formulations relying on solvent casting, where the interactions may appear at the molecular level, either in the formulation mixing stage or the evaporation stage during film formation. When the film contains a drug in its amorphous form and if the drug concentration surpasses the limit of its solubility in a polymer, crystal growth might occur, with subsequent recrystallization during storage [22]. Thus, DSC and FT-IR are important for confirming drug-excipient compatibility, as well as thermodynamic drug activity and, subsequently, the physical stability of delivery systems. Furthermore, this set of techniques may provide additional information on the FFS metamorphosis process.

**Figure 1:**
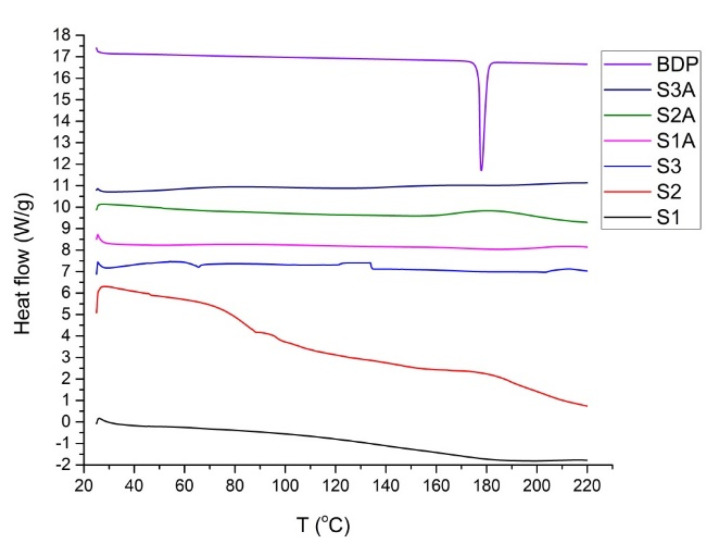
DSC scans of pure BDP and films generated from both drug-loaded and unloaded samples.

BDP showed a distinct melting endotherm peak at 178 °C. Considering the absence of this peak in thermograms of BDP-containing films (Figure 1), it can be concluded that the drug is dissolved and uniformly distributed throughout the dispersion/polymeric matrix in its molecular form. As explained by Edwards et al. [4], this is observed as a reduction in the obtained melting enthalpies. Expectedly, all the films started to melt at lower points when compared to their pure ingredients, which is partly attributed to differences in the specific film surface areas available for testing [23].

The only BDP-loaded vs. unloaded DSC curves that deserve attention are those attributed to samples S2A and S2. In case of the film generated by sample S2, a gradual heat flow decrease is apparent (Figure 1). The fact that the S2A scan does not follow the same trend but implies a broad peak approximately at the site of the BDP melting endotherm reveals the need for additional in-depth film morphology investigation (discussed later in Section 2.3, which is dedicated to the AFM technique).

Drug–polymer interactions at a molecular level are essential to reveal the mechanism of drug release and drug stability, especially in dynamic systems, such as FFSs [24]. FT-IR spectra were thus used to evaluate the possible molecular interactions between the drug, the film-forming polymer(s) and other excipients [25]. After analyzing FT-IR spectra of all relevant components, characteristic absorption bands were noted. Similar to the findings of Garcia-Couce et al. [25], for BDP, our focus was placed on bifurcated signals corresponding to C=O stretching vibrations (contribution of the carbonyl group of the chain and the ketonic group of the first ring in the characteristic cortisol structure at 1727 and 1659 cm^−1^, respectively), as well as the asymmetric stretching vibrations of –C-O [25]. The weaker signals spanning from 976 to 607 cm^−1^ attributed to *p*-O stretching and C-C vibrations in the BDP structure were put aside, as they were masked by the signals of other ingredients of the samples.

**Figure 2:**
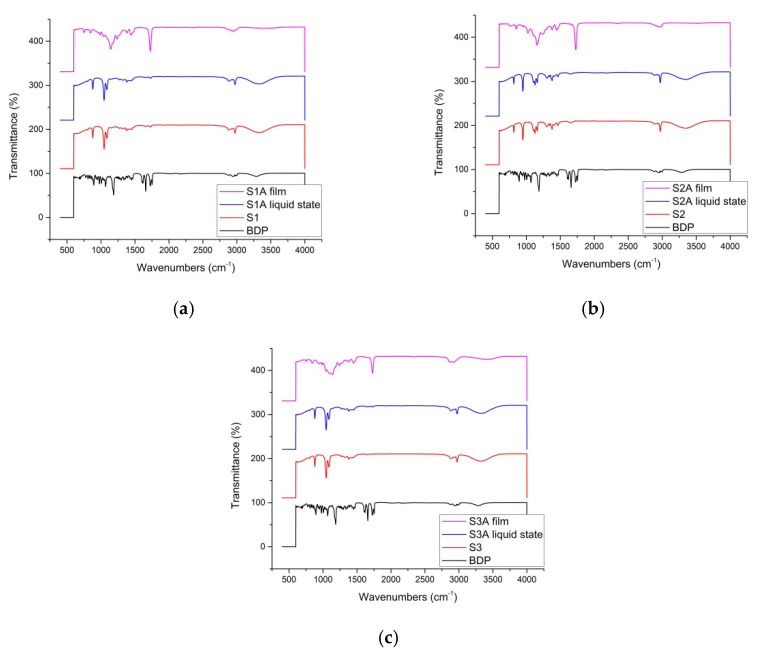
Comparative representation of FT-IR spectra obtained for BDP, ‘placebo’ FFS samples, and BDP-loaded samples in their liquid and dried (film) form provided for (**a**) samples S1/S1A, (**b**) S2/S2A and (**c**) S3/S3A.

As can be seen in Figure 2, the spectra obtained for FFS samples retained the abovementioned signals of interest for BDP as the model active substance. Within drug-loaded samples, the BDP signal initially placed at 1727 cm^−1^ merged with the signals of several compounds (namely, Eudragit^®^ RS PO at 1723 cm^−1^ (Figure 2a,c), Eudragit^®^ NE at 1731 cm^−1^ (Figure 2b) and polysorbate 80 at 1734 cm^−1^ (Figure 2a,c)) into a stronger and somewhat broader peak ranging from 1725 to 1728 cm^−1^, depending on the precise active sample. This is not surprising, considering that BDP, as the chosen model active substance, possesses a number of sites prone to hydrogen bonding. Therefore, certain interactions with BDP and OH groups of the applied film-forming polymers are inevitable, leading to the observed shift to higher wavenumbers [5]. Nevertheless, the recorded signals demonstrate preliminary stability of the BDP-loaded samples, i.e., the prevalence of physical interactions between the model drug and the present excipients [26]. These findings are of special importance for the sample characterization in the dried state, implying that the sample transformation from liquid state to the film matrix is not followed by drug precipitation but only homogeneous physical immobilization of the drug within the generated film [4].

### 2.3. AFM Analysis of the Film Surface Morphology

**Figure 3:**
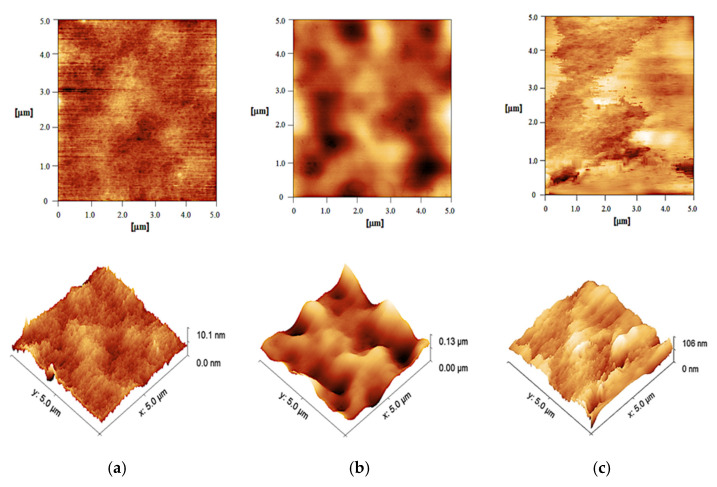
Comparative view of 2D (upper images) vs. 3D (lower) AFM images obtained by scanning the 5 × 5 μm^2^ film surface generated by the samples: (**a**) S1A, (**b**) S2A and (**c**) S3A.

AFM proved to be a valuable addition to the set of characterization methods aiming at a comprehensive QTPP of FFSs. Through AFM, it was possible to obtain a more direct insight into the formed surfaces of the selected FFSs and their homogeneity, representing one of the most important features responsible for appropriate skin contact. Figure 3 shows 2D and 3D topography images of the generated films at the nano- and microscale, enabling observation of the fine structures and morphological properties of the films. Even at first sight, three different types of polymer matrices with incorporated BDP were observed. 2D micrography of the film generated by sample S1A indicated its transparency and homogeneity, presumably with uniform drug distribution within the polymer matrix (Figure 3a). On the other hand, change in the polymer type in the S2A, compared to the S1A film, resulted in a completely different morphology (Figure 3b), which was in line with the organoleptic properties of the dried S2A sample (Table 3), as well as the DSC scans (Figure 1). There were regions that seemed to be ‘immersed’, leading to the formation of ‘valley-like’ inclusions. BDP is most likely deposited within these inclusions enclosed by polymer layers. Similar findings were observed by other authors in investigations of Eudragit-based FFSs [27]. Such structural organization could potentially lead to immediate and enhanced drug release, resulting in the subsequent formation of drug reservoirs both within the film and the stratum corneum, enabling prolonged dermal drug delivery [28].

Finally, incorporation of the HPC in S3A appears to be responsible for the undulant film surface (Figure 3c) due to the characteristic swelling of this hydrophilic polymer.

**Figure 4:**
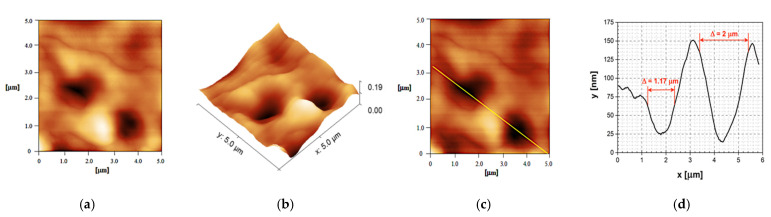
AFM images of the film topography obtained by scanning the 5 × 5 μm^2^ film surface of sample S2A (**a**), 2D and (**b**) 3D; (**c**) direction of the profile observed along the two inclusions (S2A); (**d**) profile along the two inclusions or valleys with the diameter of the inclusions/valleys.

However, the observed differences among the formulations could also be attributed to the presence of the plasticizer and its concentration within the sample, not only to the polymer used [29]. Obviously, the sample deprived of plasticizer addition (S2A) differs significantly from the other two FFSs. This observation was confirmed by analyzing the roughness of the surface. In the case of S1A, surface roughness of the polymeric film is approximately 1.0 nm, with a root mean square roughness (Rq) of 1.19 ± 0.12 nm. On the other hand, quite unexpectedly, the film formed by S2A, which contains the polymer Eudragit^®^ NE 30D, gave higher values of film surface roughness (Rq = 64.9 ± 6.7 nm) (Figure 4a,b). This could be explained by preserved integrity of the polymer (colloid) particles upon film formation, even after 24 h at 30 °C (Figure 4b). In the case of the last examined FFS formulation, S3A, the presence of polymer HPC (present in a concentration of 1% and combined with Eudragit^®^ RS PO, 4%), when compared to S1A (exclusively containing Eudragit^®^ RS PO) increased the scale of film roughness to 19.4 nm. Therefore, incorporation of the hydrophilic polymer in S3A followed by a lower plasticizer concentration (1% in S1A vs. 0.5% in S3A) appears to be responsible for the wavy film surface (Figure 3c), resulting in larger-scale structures contrary to hydrophobic film-former Eudragit^®^ RS PO, which provided a smooth surface of the films generated by S1A (Figure 3a).

It can be concluded that the conformational structure of the polymer has an important influence on the higher film surface roughness attributed to the polymer Eudragit^®^ NE 30D compared to the film surface with the polymer Eudragit^®^ RS PO. This refers to the formations of large-diameter inclusions (troughs) on the surface of the film formed by sample S2A. Figure 4c represents a direction of an imaging profile along the two inclusions (troughs), whereas Figure 4d marks a depth of formed troughs ranging from 50 to 135 nm, with trough diameters in the range of 1.17–2 μm.

AFM confirmed that the levels of roughness/smoothness of the film surfaces were different between the tested samples, mostly depending on the type and morphological characteristics of the polymer present in the composition of the FFS formulation (hydrophobic polymers Eudragit^®^ RS PO or Eudragit^®^ NE 30D vs. the hydrophilic polymer HPC) but also depending on the presence of a plasticizer.

The results of the tribology study published in our previous paper [15] correlate well with the abovementioned AFM-mediated observations. Smoother films require less force from the Frictiometer probe, resulting in lower friction values and vice-versa. Therefore, the sample based on the hydrophobic neutral polymer Eudragit^®^ NE 30D exhibited different tribological behavior than the other samples by maintaining relatively high friction throughout the experiment. This was confirmed by AFM, correlating skin friction with the actual film surface roughness; the roughness of the film surface is inversely correlated with the friction value. It is also interesting to note that the results of the performed physicomechanical testing (presented in the Section 2.1) were also in line with the results obtained with the AFM technique, confirming the best film resistance (folding endurance) of the samples containing the cationic polymer Eudragit^®^ RS PO, either alone or in combination with HPC.

### 2.4. In Vitro Permeation of BDP through the Porcine Ear Epidermis

**Figure 5:**
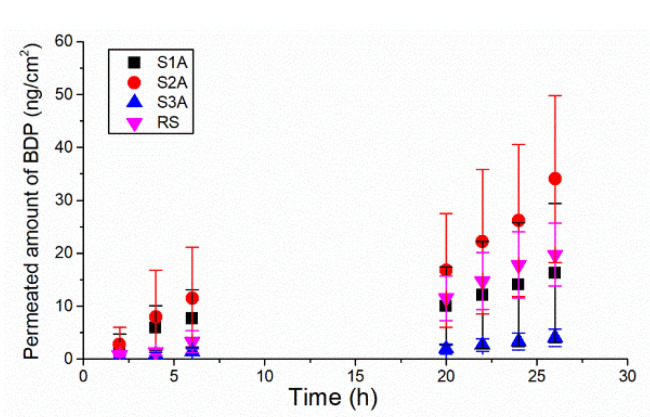
In vitro permeation profiles of BDP determined across the heat-separated porcine ear epidermis (mean ± SD, *n* = 5) reflecting the influence of differences in formulation composition of the investigated samples on in vitro BDP skin absorption.

In this part of the study, in vitro permeation of the selected model drug BDP from three different types of polymeric FFSs was investigated and compared with commercially available betamethasone ointment 0.5 mg/g (hereafter labelled as the reference sample, RS) under an infinite-dose regimen in order to explore the capability of polymeric films to determine the permeation rates suitable for topical therapy. Likewise, it was intriguing to evaluate how different film-forming formulations containing either hydrophobic Eudragit^®^ RS PO (8.5%), Eudragit^®^ NE 30D (6.0%) polymer or a combination of hydrophobic Eudragit^®^ RS PO (4%) and hydrophilic HPC (1%) polymers in substandard total polymer concentration affect BDP delivery though the porcine ear epidermis. The obtained cumulative permeation profiles of BDP and corresponding in vitro permeation parameters are presented in Figure 5 and Table 4, respectively.

The obtained permeation profiles clearly indicated differences in BDP permeation among the tested formulations. Based on the total quantity of BDP permeated through the heat-separated pig ear epidermis after 26 h (Table 4), the investigated formulations were ranked in the following order: S2A > S1A > RS > S3A. Strictly considering the results obtained for different FFSs, it is obvious that the formulation composition (type of polymer, solvent and plasticizers used for sample preparation) significantly influenced the BDP permeation profiles. In particular, as seen in Figure 5, sample S2A, prepared with the hydrophobic polymer Eudragit^®^ NE 30D, showed the highest quantity of BDP permeated in 26 h, as well as the highest value of steady-state flux and permeation coefficient (Table 4). On other hand, a trend of lower permeated amount of BDP over 26 h (particularly in the latter time points), including statistically lower values of steady-state flux and permeation coefficient (*p* < 0.05, *t*-test), was observed for S1A formulation containing Eudragit^®^ RS PO when compared with sample S2A. However, this finding cannot be ascribed to variation of the polymer type within sample preparation but rather to the presence of plasticizers within the S1A formulation. Taking into account the study reported by Gennari et al. [30], it appears that plasticizers (polysorbate 80 and propylene glycol) reduced the thermodynamic activity of BDP by improving its solubilization within the film, leading to a decreased BDP flux through the epidermal membrane.

Interestingly, the addition of hydrophilic HPC polymer (Klucel^®^ GF) in the formulation containing the hydrophobic polymer (Eudragit^®^ RS PO) and plasticizers (polysorbate 80 and propylene glycol) (i.e., sample S3A) further decreased the permeation of BDP through the epidermal membrane, although no statistically significant difference in the monitored permeation parameters was found (*p* > 0.05, Mann Whitney U test) compared to the S1A formulation (Table 4). There are several factors that could contribute to the reduced BDP permeation from this sample. Firstly, sample S3A, as the most viscous tested formulation, is associated with the highest film thickness (Table 4); consequently, decreased BDP uptake through the epidermal membrane could derivate from limited drug diffusivity, i.e., drug release from the film. Likewise, the results of a study reported by Garvie-Cook and colleagues should be noted [29], which demonstrated the superior skin penetration of lipophilic betamethasone-17-valerate from FFS formed with hydrophobic polymers, specifically Eudragit^®^ RS PO and Dermacryl^®^ 79, relative to those prepared with the more hydrophilic Klucel^TM^ LF, due to, at least in part, the superior anti-nucleation efficiency of the former. During solvent evaporation, it was suggested that these hydrophobic polymers may be able to stabilize the drug in a transient, supersaturated state better than Klucel^TM^ LF by hydrogen bond interactions between the drug and the H-bond-accepting amine groups. As a result, this enables the establishment of drug ‘reservoirs’ within both the residual polymeric film on the skin surface and the stratum corneum, from which the drug then may diffuse continuously over time into the lower, viable skin layers and the receptor compartment [27,29]. The described phenomenon could also support the observed, significantly improved permeation of BDP from sample S2A, which was exclusively prepared with Eudragit^®^ NE 30D polymer.

Finally, when comparing the tested FFS with the selected reference formulation (Beloderm^®^ ointment), a statistically significant difference in all monitored permeation parameters was observed between the RS and S2A formulations, as well as RS and S3A, without remarkable differences between the RS and S1A formulations (*p* < 0.05, *t*-test or Mann Whitney U test). The limited permeability of BDP makes the S3A formulation a promising candidate for sustained skin delivery of BDP. In particular, the observed slow permeation profile could enable a reduction in administration frequency, leading to improved patient treatment compliance. Simultaneously, owing to the highest skin substantivity (previously demonstrated for corresponding placebo sample [15]), this formulation could be particularly suitable for drug administration on high-friction body areas. However, further in vivo studies are needed to determine whether the obtained findings may have clinical relevance and significant influence on BDP therapeutic efficacy.

## 3. Materials and Methods

### 3.1. Materials

Betamethasone dipropionate (BDP), the model active pharmaceutical ingredient, was kindly donated by Galenika (Belgrade, Serbia). The drug reference product used for in vitro permeation study was Beloderm^®^ ointment, purchased from Belupo (Zagreb, Croatia). Hydrophilic polymer Klucel^®^ GF (hydroxypropyl cellulose) was supplied by Caesar & Loretz GmbH (Hilden, Germany). Hydrophobic polymethacrylate copolymers Eudragit^®^ NE 30D (poly(ethyl acrylate-co-methyl methacrylate) 2:1 (polyacrylate dispersion 30% Ph. Eur.)) and Eudragit^®^ RS PO (poly(ethyl acrylate-co-methyl methacrylate-co-trimethylammonioethyl methacrylate chloride), i.e., copolymer type B, Ph. Eur.) were obtained from Evonik Rohm GmbH (Darmstadt, Germany). Polysorbate 80 (Sigma Aldrich, Schnelldorf, Germany), glycerol, propylene glycol and solvents used (all Carl Roth GmbH, Karlsruhe, Germany) were of pharmaceutical grade. For HPLC-MS/MS analysis, acetonitrile LC-MS grade (J.T. Baker, Phillipsburg, NJ, USA), formic acid (Acros Organics, Geel, Belgium) and deionized water (Gen Pure Ultrapure, Thermo Fisher Scientific GmbH, Dreieich, Hessen, Germany) were used.

### 3.2. Preparation Method of the Polymeric FFSs with a Lipophilic Model Drug

The samples were prepared according to the compositions specified in Table 1. The content of BDP was set to suit its therapeutic concentration of 0.064%, corresponding to 0.05% betamethasone. In a closed vessel, BDP (previously measured on an ABJ 120–4 M analytical scale; Kern & Sohn GmbH, Balingen, Germany) was dissolved in a small amount of the selected solvent (ethanol 96% (*v*/*v*) or isopropyl alcohol), wherein the addition of plasticizer (propylene glycol) depended on the chosen type of the polymer (Eudragit^®^ RS, HPC or their combination) and, optionally, the addition of the surfactant/potential penetration enhancer (polysorbate 80), while stirring continuously until the drug was completely dissolved. The chosen polymer or a combination of polymers was gradually dissolved/dispersed in the residual amount of the selected solvent(s) and a smaller amount of water while stirring continuously with a magnetic stirrer (RCT basic, IKA, Staufen, Germany). After 2.5–3 h of mixing, the pre-dissolved drug was added to the polymer dispersion. The mixing was continued on the magnetic stirrer up to 24 h, until complete homogenization. All formulations were stored in tightly closed dark glass vials.

### 3.3. Characterization of Physicochemical, Mechanical and Sensory Properties

#### 3.3.1. Assessment of Drying Time, Thickness, Spreadability, Flexibility and Stickiness of the Casted Films

In order to assess the full impact of BDP incorporation into FFS, the samples were subjected to a series of specifically optimized characterization methods in order to screen for the following properties of the generated films: drying time, thickness, spreadability, flexibility and basic sensory properties. This allowed for comparison of the obtained numerical and descriptive values with the results attributed to placebo samples published in our previous study [15]. All the parameters were assessed in triplicate in order to express the results in the form of a mean value ± standard deviation (SD) whenever applicable.

Drying time of the films casted by 500 μL of each sample was measured with a stopwatch, both at room (23 ± 2 °C) and skin temperature (32 ± 0.1 °C; Orbital Shaker Incubator ES 20, Biosan, Riga, Latvia) by touching the film surface. The obtained mean drying times were expressed in min ± SD and graded as low (≤5 min), medium (5–7 min) or high (>7 min), according to [31].

Film thickness was assessed with a digital micrometer compliant with DIN 863, supporting a range of thickness measurements of 0–25 mm/0.001 mm (Kern, Balingen, Germany), by applying 10 μL of the samples to a microscope glass plate. After complete drying, the thickness of the film was measured and corrected to the thickness of the clean substrate, resulting in mm ± SD.

Film spreadability was evaluated via film surface measurement by applying 10 μL of each sample perpendicularly to the surface of a microscope glass plate. After complete film drying, the diffusion area was determined using a millimeter graph paper and expressed in mm^2^ ± SD.

Flexibility (key attribute of a film’s mechanical resistance) was evaluated with the so-called folding technique. The folding endurance value is defined as the number of times a film can be folded at the same place without breaking. Hence, the lower the folding endurance value, the more brittle the film is, allowing the test to assume a film’s integrity [26,32]. A volume of 500 μL of each sample was uniformly distributed over a length of 4 cm onto a 27 × 4.4 cm rubber substrate. After the film dried, a rubber band was repeatedly rolled and unrolled over the whole length of the film. After each rolling cycle, the film was inspected for changes using a magnifying glass. The resulting number of folds (i.e., the folding endurance value) was expressed as the number of rolling times up to the first perceived, visible change of the film on the rubber substrate ±SD.

The visual appearance of the formed films was descriptively appraised in terms of color, transparency, structure, homogeneity, gloss and stickiness. Film stickiness was additionally evaluated by applying a piece of cotton bud to the dry film [31]. The cotton bud was weighed on an analytical balance (ABJ 120–4 M, Kern & Sohn GmbH, Balingen, Germany) both before and after application, which allowed the stickiness to be rated as ‘low’ (no observable cotton fibers on the film surface), ‘medium’ (a thin layer of fiber filaments is visible, with 0.01 g as the cutoff value of the retained cotton) or ‘high’ (0.02 g as the cutoff value of the retained cotton).

##### 3.3.2. pH Values of FFS

Measurement of FFS pH values was performed by direct immersion of the previously calibrated pH checker^®^ HI98103 probe (Hanna Instruments, Woonsocket, RI, USA). Testing was carried out initially (24 h after sample preparation), as well as after 3 and 6 months of storage at room temperature, as a manner of stability screening. All the measurements were performed in triplicate, resulting in mean values of three individual measurements (±SD).

#### 3.3.3. Viscosity Measurements

Rheological profiles of drug-loaded FFSs and their respective placebo samples were assessed with an Anton Paar MCR 302 rheometer with RheoCompass software (Anton Paar GmbH, Graz, Austria). A measuring tool for low-viscosity and viscoelastic liquids was used (Concentric cylinder CC27), and the measurements were performed at 20 ± 0.1 °C. Average viscosity at different shear rates was considered important for both sample characterization and stability evaluation. Due to the volatility of FFSs, as a manner of stability control, viscosity was assessed initially, as well as after 3 and 6 months of storage at room temperature.

### 3.4. Morphology and Methamorphosis Characterization

#### 3.4.1. Differential Scanning Calorimetry (DSC)

After assessing thermal changes of BD and relevant excipients used for sample preparation (namely, film-forming polymers, polysorbate 80 and propylene glycol), DSC was performed on the samples. Considering the volatile nature of the investigated FFSs, measurements commenced after complete drying of the generated films (the samples were casted into aluminum pans and left overnight to allow for complete solvent evaporation). Depending on the nature of the formulation, 2 to 10 mg of the dried samples were heated from 25 to 220 °C, with a heating rate of 10 °C/min, using DSC 1 (Mettler-Toledo GmbH, Greifensee, Switzerland). The nitrogen flow was set to 50 mL/min.

#### 3.4.2. Fourier Transform Infrared Spectroscopy (FT-IR) Analysis

The attenuated total reflectance FT-IR spectra were recorded using a Nicolet iS10 FT-IR spectrometer (Thermo Fisher Scientific, Horsham, UK) in the wavelength range between 4000 and 600 cm^−1^ with a resolution of 4 cm^−1^. After obtaining the spectra of all relevant substances (Table 1), the samples were analyzed both in liquid and dried state in order to assess possible drug–matrix interactions, as well as transformation phenomena of the FFS. Peak matching was performed to detect any possible interactions between the components.

#### 3.4.3. Atomic Force Microscopy

In order to investigate the 3D structure and the homogeneity of the polymeric film, AFM analysis of the samples was performed by applying an NTEGRA Prima atomic force microscope (NT-MDT, Moscow, Russia). Intermittent-contact AFM mode was applied using NT-MDT NSGO1 silicon, N-type, antimony doped cantilevers with Au reflective coating. The nominal force constant of the cantilevers was 5.1 N/m, whereas the cantilever driving frequency was around 150 kHz. AFM images were created and analyzed with Image Analysis 2.2.0 (NT-MDT) and Gwyddion 2.60 (Free and Open Source software, Czech Metrology Institute, Jihlava, Czechia) software. Prior to actual measurement, the samples were cast on mica plates and left overnight at 30 °C.

### 3.5. In Vitro Skin Permeation Study

In vitro experiments were conducted under infinite dosing conditions using modified Franz diffusion cells (Gauer Glas, D-Püttlingen, Germany) with an effective diffusion area of 2.01 cm^2^ and a receptor volume of 12 mL, with a porcine ear epidermis (heat-separated) as a membrane. The epidermal membrane was carefully prepared according to the previously described protocol [33]. In brief, fresh porcine ears obtained from a local abattoir immediately after slaughter were washed under cold running water, and skin of the external side was carefully excised using a scalpel. The isolated skin was then cleaned off with isotonic saline and cotton swabs, blotted dry with a soft tissue, wrapped in aluminum foil and stored at −20 °C (within one month). On the day of the experiment, after thawing at room temperature, the skin was punched to the discs with a diameter 25 mm. Subsequently, the skin pieces were immersed in water at 60 °C for 90 s, and the SC-viable epidermis layer was separated from the underlying dermis using forceps. The isolated epidermal sheets were transferred to Petri dishes filled with phosphate-buffered saline (PBS; pH = 7.4) until use (within 1 h).

The heat-separated epidermal membranes were mounted between donor and receptor chambers filled with degassed, pre-heated (32 °C) receptor medium (a mixture of PBS and ethanol (96%) at a ratio 50:50 *v*/*v*). Afterwards, receptor parts of the cells were immersed in a water bath at 32 ± 1 °C in order to equilibrate the membranes under constant magnet stirring at 500 rpm. After 30 min, 300 μL of each of investigated FFS formulation (S1A, S2A, S3A) was uniformly applied to the membrane surface. Commercial product betamethasone ointment, 0.5 mg/g (Beloderm^®^ ointment, Belupo, Zagreb, Croatia) was used as a drug reference sample (1 g of each FFS/1 g of Beloderm^®^ ointment contains 0.64 mg BDP). Drug permeation through the heat-separated porcine ear epidermis was observed over the 26 h period to achieve a steady state, as well as to avoid inevitable degradation of the skin barrier. The temperature (32 ± 1 °C) and stirring speed (500 rpm) were maintained constant. At seven previously defined time points (2 h, 4 h, 6 h, 20 h, 22 h, 24 h and 26 h), 600 μL aliquots were withdrawn and immediately replaced with an equal volume of fresh, pre-heated receptor medium in order to maintain sink conditions. BDP concentration in the sampled aliquots was then assayed by the HPLC–MS/MS method. In order to fully characterize the permeation process, the cumulative amount of BDP permeation per unit area (ng/cm^2^) was plotted against time (*t*), and then the permeation rate (steady state flux) was determined from the slope of the linear portion of the plot for each investigated formulation. Permeation coefficients (ng/cm^2^ h) were calculated by dividing permeation rates (ng/cm^2^ h) by the initial concentration of BDP in the vehicle (ng/mg).

### 3.6. HPLC-MS/MS

BDP in aliquots obtained during in vitro permeation study was determined by the HPLC-MS/MS method. A liquid chromatographic system, Accela 1000 (Thermo Fisher Scientific, San Jose, CA, USA), consisting of an auto sampler and quaternary pump, was used. Chromatographic separation was achieved using a ZORBAX Eclipse Plus C8 column (150 mm × 4.6 mm, 5 µm; Agilent Technologies, Santa Clara, CA, USA) at 40 °C. A mixture of acetonitrile and 0.1% formic acid (80:20, *v*/*v*) was used for isocratic elution at a flow rate of 500 µL/min. The total analysis time was 6.5 min. Mass analyses were conducted on a TSQ Quantum Access MAX triple-quadrupole spectrometer equipped with a heated electrospray ionization (HESI) source, with high-purity nitrogen as nebulizing gas. HESI source parameters were optimized by syringe infusion (20 µL/min) of BDP standard solution. HESI source and mass spectrometry parameters were as follows: spray voltage, 5000 V; vaporizer temperature, 350 °C; sheath gas pressure, 30 units; ion sweep gas, 0 units; auxiliary gas, 10 units; ion transfer capillary temperature, 250 °C; capillary offset, 35 units; tube lens offset, 136 units; skimmer offset, 36 units; scan width (*m*/*z*), 0.02; scan time, 200 ms. Mass spectrometry was used to detect specific ions for analyte identification. Detection of the ions was performed in the selected reaction monitoring (SRM) positive scan mode using a transition of *m*/*z* 527.3 → 453.2 (collision energy was 20 V). Xcalibur software (Thermo Fisher Scientific, San Jose, CA, USA) was used for data acquisition and processing.

### 3.7. Statistical Analysis

Where applicable, data are presented as mean ± SD. Statistical analysis was performed using Student’s *t*-test or one-way analysis of variance (ANOVA), followed by a Tukey post hoc test or by a nonparametric Kruskal–Wallis test and by a Mann–Whitney U test for pairwise comparisons, depending on the nature of the data. An assessment of the normality of data was carried out using the Shapiro–Wilk test. Statistical analyses were performed using the PASW Statistics software package, version 18.0 (SPSS Inc., Chicago, IL, USA). The level of significance was set to *p* < 0.05.

## 4. Conclusions

FFSs are dynamic dosage forms with challenging characterization. Considering the possible therapeutic advantages these systems may convey to a modern patient, FFS development and evaluation should be rationally defined. The present study revealed the utility of several methods able to refine the number of needed tests within the final QTPP. In this regard, apart from the viscosity and pH value, physicomechanical properties (namely, film drying time, surface, folding endurance and thickness) are identified as important CQAs of FFSs because they directly affect ease of administration, skin retention, product performance and patient acceptability. Coupling AFM, DSC and FT-IR proved to provide a deeper insight into the mechanisms of metamorphosis/transformation of FFSs (e.g., absence of drug precipitation upon film generation while providing drug reservoirs), enabling a reduction in the number of in vitro biopharmaceutical and in vivo skin performance tests.

Thermal and spectroscopic characterization implied that BDP remains stable throughout the liquid-to-film transition in the investigated systems, comprising hydrophobic and/or hydrophilic polymers. At the 2D level, AFM depicts film homogeneity, whereas a 3D view exposes in-depth film morphology in the context of, among other factors, the presence and positioning of drug reservoirs. DSC contributes to confirmation of drug solubility (i.e., absence of drug precipitation) in the generated polymeric film. Therefore, by coupling these methods, tailored FFSs can be efficiently characterized in an early stage of development. In our study, various characterization stages (e.g., FFS viscosity, film thickness, ultimately confirmed in our permeation study) implied that the sample with a combination of film-forming polymers (Eudragit^®^ RS PO and HPC) showed potential for sustained drug delivery. On the other hand, the Eudragit^®^ NE 30D-based FFS increased the rate and extent of BDP permeation. The observed enhancement could be connected with the presence of valleys (inclusions) discovered by AFM. Naturally, only clinical studies would reveal more details about the treatment efficacy of such systems targeting psoriatic lesions; however, establishing a drug reservoir in the residual polymeric film could enable both sustained and enhanced skin delivery.

## Figures and Tables

**Table 1:** Composition of the selected in situ film-forming formulations loaded with the model active pharmaceutical ingredient, BDP.

Sample Composition (%, m/m)	S1A	S2A	S3A
Betamethasone dipropionate	0.064	0.064	0.064
Eudragit^®^ RS PO	8.5	-	4.0
Eudragit^®^ NE 30D	-	6.0	-
Klucel^®^ GF	-	-	1.0
Propylene glycol	1.0	-	0.5
Ethanol, 96% (*v*/*v*)	86.7	-	92.9
Isopropyl alcohol	-	85.0	-
Polysorbate 80	1.0	-	0.3
Water, purified	up to 100	up to 100	up to 100

**Table 2:** Viscosity and pH values of the BDP-loaded FFS over 6 months of storage at room temperature (mean ± SD, *n* = 3).

Sample	Time Point	Viscosity (mPa·s)	pH Value
S1A	Initially	1.67 ± 0.03	7.1 ± 0.1
	After 3 months	1.77 ± 0.20	6.8 ± 0.2
	After 6 months	1.74 ± 0.16	6.6 ± 0.1
S2A	Initially	26.10 ± 0.62	7.7 ± 0.1
	After 3 months	25.16 ± 0.63	7.5 ± 0.1
	After 6 months	24.54 ± 0.62	7.3 ± 0.1
S3A	Initially	47.17 ± 3.06	7.0 ± 0.2
	After 3 months	49.07 ± 3.49	6.8 ± 0.1
	After 6 months	47.32 ± 3.16	6.7 ± 0.1

**Table 3:** Physicomechanical evaluation of the BDP-loaded film-forming systems (mean ± SD, *n* = 3).

Sample	Film’s Organoleptic Appearance	Film-Drying Time at 32.0 ± 0.1 °C (min)	Film-Drying Time at 25 ± 2 °C (min)	Film Surface (mm^2^)	Folding Endurance Value	Film Thickness (mm)
S1A	Colorless, transparent, structured, homogenous, glossy, low stickiness	6.6 ± 0.1 ^a^	24.0 ± 0.6 ^c^	210.0 ± 14.2 ^c^	111.0 ± 2.0	0.007 ± 0.001 ^c^
S2A	Colorless, mildly turbid, fine-structured, homogenous, low stickiness	6.3 ± 0.1	47.0 ± 0.6 ^c^	77.0 ± 1.0 ^c^	54.0 ± 2.0 ^d^	0.015 ± 0.002 ^c^
S3A	Whitish, mildly turbid, honeycomb-like structure, homogenous, matt, low stickiness	5.0 ± 0.1 ^b^	31.0 ± 1.2 ^c^	84.0 ± 3.0 ^c^	107.0 ± 1.0	0.032 ± 0.006 ^c^

^a^ *p* < 0.05 compared to S3A; ^b^ *p* < 0.05 compared to S1A; ^c^ *p* < 0.05 compared to all tested formulations; ^d^ *p* < 0.05 compared to S1A and S3A.

**Table 4:** Permeation parameters of BDP from tested film-forming formulations (S1A, S2A and S3A) and reference betamethasone ointment 0.5 mg/g (RS) obtained using porcine ear epidermis as the membrane (mean ± SD, *n* = 5).

Sample	Permeation Rate (ng/cm^2^ h)	Q_26 h_ * (ng/cm^2^)	Permeation Coefficient (mg/cm^2^ h)
S1A	1.039 ± 0.783	16.277 ± 13.130	0.0016 ± 0.0012
S2A	2.964 ± 0.851 ^a^	34.049 ± 15.795 ^a^	0.0046 ± 0.0013 ^a^
S3A	0.348 ± 0.157 ^b^	4.026 ± 1.615 ^b^	0.0005 ± 0.0002 ^b^
RS	1.247 ± 0.209	19.726 ± 5.953	0.0019 ± 0.0003

* Amount of BDP permeated across the pig ear epidermis after 26 h. ^a^ *p* < 0.05 compared to S1A, S3A and RS. ^b^ *p* < 0.05 compared to RS.

## Data Availability

Not applicable.

## References

[1] Tan X., Feldman S.R., Chang J., Balkrishnan R. (2012). Topical drug delivery systems in dermatology: A review of patient adherence issues. Expert Opin. Drug Deliv..

[2] Rottke M., Lunter D.J., Daniels R. (2014). In vitro studies on release and skin permeation of nonivamide from novel oil-in-oil-emulsions. Eur. J. Pharm. Biopharm..

[3] Kathe K., Kathpalia H. (2017). Film forming systems for topical and transdermal drug delivery. Asian J. Pharm. Sci..

[4] Edwards A., Qi S., Liu F., Brown M.B., McAuley W.J. (2017). Rationalising polymer selection for supersaturated film forming systems produced by an aerosol spray for the transdermal delivery of methylphenidate. Eur. J. Pharm. Biopharm..

[5] Hifumi H., Ewing A.V., Kazarian S.G. (2016). ATR-FTIR spectroscopic imaging to study the drying and dissolution of pharmaceutical polymer-based films. Int. J. Pharm..

[6] Liqui-Patch®. https://epinamics.com/the-liqui-patch.

[7] MedSpray®. https://www.medpharm.com/us/expertise/routes-of-delivery/.

[8] Durapeel®. https://www.crescitatherapeutics.com/durapeel.

[9] Estradiol MTDS. https://www.acrux.com.au/product/estradiol-mdts/.

[10] Axiron®. https://www.acrux.com.au/product/testosterone-solution/.

[11] Lamisil® Once 1% Cutaneous Solution. https://www.medicines.org.uk/emc/product/179/smpc#gref.

[12] Kis N., Kovács A., Budai-Szűcs M., Gácsi A., Csányi E., Csóka I., Berkó S. (2019). Investigation of silicone-containing semisolid in situ film-forming systems using QbD tools. Pharmaceutics.

[13] Kovács A., Kis N., Budai-Szűcs M., Gácsi A., Csányi E., Csóka I., Berkó S. (2020). QbD-Based Investigation of Dermal Semisolid in situ Film-Forming Systems for Local Anaesthesia. Drug Des. Devel. Ther..

[14] [EMA] European Medicines Agency (2018). CHMP/QWP/708282/2018 Draft Guideline on Quality and Equivalence of Topical Products.

[15] Timotijević M.D., Ilić T., Savić S., Pantelić I. (2022). Simultaneous physico-mechanical and in vivo assessment towards factual skin performance profile of topical polymeric film-forming systems. Pharmaceutics.

[16] Buechler C.R., Veenstra J., Gold L.S. (2021). New topical therapies for psoriasis. Dermatol. Rev..

[17] Horn E.J., Domm S., Katz H.I., Lebwohl M., Mrowietz U., Kragballe K. (2010). Topical corticosteroids in psoriasis: Strategies for improving safety. JEADV.

[18] Psomadakis C.E., Han G. (2019). New and emerging topical therapies for psoriasis and atopic dermatitis. J. Clin. Aesthet. Dermatol..

[19] Hadgraft J., Lane M.E. (2016). Drug crystallization-implications for topical and transdermal delivery. Expert Opin. Drug Deliv..

[20] Parhi R., Goli V.V.N. (2020). Design and optimization of film-forming gel of etoricoxib using research surface methodology. Drug Deliv. Transl. Res..

[21] Van Bocxlaer K., McArthur K.-N., Harris A., Alavijeh M., Braillard S., Mowbray C.E., Croft S.L. (2021). Film-Forming Systems for the Delivery of DNDI-0690 to Treat Cutaneous Leishmaniasis. Pharmaceutics.

[22] Woertz C., Kleinebudde P. (2015). Development of orodispersible polymer films containing poorly water soluble active pharmaceutical ingredients with focus on different drug loadings and storage stability. Int. J. Pharm..

[23] Hanna P.A., Ghorab M.M., Gad S. (2019). Development of betamethasone dipropionate-loaded nanostructured lipid carriers for topical and transdermal delivery. Antiinflamm. Antiallergy Agents Med. Chem..

[24] Tran T.T.D., Tran P.H.L. (2020). Molecular interactions in solid dispersions of poorly water-soluble drugs. Pharmaceutics.

[25] García-Couce J., Vernhes M., Bada N., Agüero L., Valdés O., Alvarez-Barreto J., Fuentes G., Almirall A., Cruz L.J. (2021). Synthesis and Evaluation of AlgNa-g-Poly(QCL-co-HEMA) Hydrogels as Platform for Chondrocyte Proliferation and Controlled Release of Betamethasone. Int. J. Mol. Sci..

[26] Zayed G.M., Rasoul S.A., Ibrahim M.A., Saddik M.S., Alshora D.H. (2020). In vitro and in vivo characterization of domperidone-loaded fast dissolving buccal films. Saudi Pharm. J..

[27] Garvie-Cook H., Frederiksen K., Petersson K., Guy R.H., Gordeev S. (2015). Characterization of topical film-forming systems using atomic force microscopy and Raman microspectroscopy. Mol. Pharm..

[28] Pünnel L.C., Lunter D.J. (2021). Film-forming systems for dermal drug delivery. Pharmaceutics.

[29] Garvie-Cook H., Frederiksen K., Petersson K., Guy R.H., Gordeev S.N. (2015). Biophysical elucidation of the mechanism of enhanced drug release and topical delivery from polymeric film-forming systems. J. Control. Release.

[30] Gennari C.G.M., Semin F., Franzè S., Musazzi U.M., Quaroni G.M.G., Casiraghi A., Cilurzo F. (2017). A glimpse in critical attributes to design cutaneous film forming systems based on ammonium methacrylate. J. Drug Deliv. Sci. Technol..

[31] Zurdo Schroeder I., Franke P., Schaefer U.F., Lehr C.M. (2007). Development and characterization of film forming polymeric solutions for skin drug delivery. Eur. J. Pharm. Biopharm..

[32] Aqil M., Ali A., Sultana Y., Najmi A.K. (2004). Fabrication and evaluation of polymeric films for transdermal delivery of pinacidil. Pharmazie.

[33] Ilić T., Pantelić I., Lunter D., Đorđević S., Marković B., Ranković D., Daniels R., Savić S. (2017). Critical quality attributes, in vitro release and correlated in vitro skin permeation—in vivo tape stripping collective data for demonstrating therapeutic (non)equivalence of topical semisolids: A case study of “ready-to-use” vehicles. Int. J. Pharm..

